# Supplementary material for: Identification of Freezing-Responsive microRNAs and Their Targets in Chinese Jujube by Small RNA and Degradome Sequencing
Source: Int J Mol Sci. 2026 Sep 4;27(17):7912. doi: 10.3390/ijms27177912 (PMC13566890; doi:10.3390/ijms27177912)
Supplement: Supplementary file 1 [file ijms-27-07912-s001.zip › Supplementary Material S2/novel_miR_35.pdf]

Provisional ID	:	novel_miR_35
Score total	:	0.9
Score for star read(s)	:	-1.3
Score for read counts	:	0
Score for mfe	:	0.6
Score for randfold	:	1.6
Score for cons. seed	:	
Total read count	:	79
Mature read count	:	79
Loop read count	:	0
Star read count	:	0

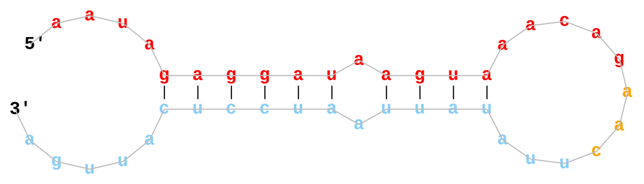

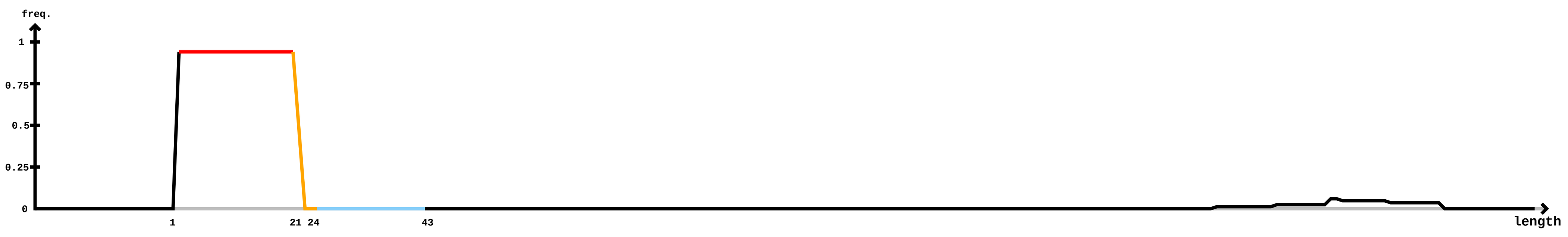

Mature Star

```
5'- cuauacaaauaaauaaccuccaaauagaggauaaguaaacagaacuuauauuaaucccucauugagcuucuuugcuauacgaucaauggguaaugaacccaggggauuguggagucauuaucguaauauacuucaucauccucaucaaacucauuuguuaaguugcuuggcagagcuggugcgauuuggcaaagcuggugugagagcuacugcaauuuggaauuuucgcaaagcuggugcgagauugugc -3'
    ................(((..(((.((((((.((((..........)))).))))))))))))...((((((((..((((...(((((.....))))).((((((.(((((((.((((...))))..)))))))).))))))....(((......)))..))))..))))))))...(((((((((.(((.((((..((((((((...(........)...)))))))).))))..))).)))))))))
```

exp	reads	mm	sample
......................aauagaggauaaguaaacag......	47	0	LCK-1
......................aauagaggauaaguaaacag......	32	0	LCK-2
......uggugCgagagcuacugcaau......	1	1	LT-1
......gcuacugGaauuuggaauu......	1	1	JCK-3
......auuuggaauuuuGgcaaag......	1	1	JCK-1
......auuuggaauuuuGgcaaag......	2	1	JCK-2
